# Phytochemistry, Pharmacology, and Traditional Medicine Applications of *Juniperus sabina* L.: A Comprehensive Overview

**DOI:** 10.3390/molecules29245876

**Published:** 2024-12-12

**Authors:** Lan Pan, Tianyuan Zhou, Chen Chen, Haiyan Xu, Wenli Wang

**Affiliations:** 1 College of Traditional Chinese Medicine, Xinjiang Medical University, Urumqi 830017, China; panlan_sc@126.com (L.P.); a18599412602@outlook.com (T.Z.); 2 Jiangsu Key Laboratory of Bioactive Natural Product Research and State Key Laboratory of Natural Medicines, School of Traditional Chinese Pharmacy, China Pharmaceutical University, Nanjing 210009, China; chenchen1601@126.com; 3 College of Pharmacy, Nantong University, Nantong 226000, China

**Keywords:** *Juniperus sabina* L., traditional uses, phytochemistry, pharmacology, toxicology

## Abstract

*Juniperus sabina* L., a species within the *Juniperus* genus, is broadly distributed across Southern Europe and Central Asia. In China, its berries, branches, and leaves are traditionally employed in Uyghur medicine to address conditions such as rheumatoid arthritis, itchy skin, tinnitus, deafness, and urinary discomfort. Notably, the plant’s primary bioactive constituents are terpenoids, lignans, and flavonoids. Contemporary pharmacological studies have revealed that *J. sabina* exhibits a range of bioactivities, including insecticidal, antimicrobial, antioxidant, anti-inflammatory, and antitumor effects. These diverse therapeutic properties can be attributed to its rich chemical profile, which comprises 279 distinct compounds such as terpenoids, lignans, flavonoids, coumarins, and others, isolated to date. This comprehensive review systematically organizes and summarizes the botanical characteristics, traditional uses, chemical composition, pharmacological activities, toxicity, limitations, and future prospects of *J. sabina*. It aims to offer a valuable scientific reference and fresh perspectives for further research, development, and application of this plant.

## 1. Introduction

The genus *Juniperus*, belonging to the Cupressaceae family, comprises approximately 70 to 80 species distributed throughout Southern Europe and Central Asia [1]. Within China, this genus is represented by fifteen species and five varieties [2] (p. 359). *Juniperus sabina* L., a low-growing shrub typically less than 1 m in height, is a member of this genus. It is predominantly dioecious, though occasionally monoecious, and is widely found in forests, rocky shrublands, hills, and sand dunes in regions such as Iran, Turkey, and China. This species holds considerable medicinal, ornamental, ecological, and economic value.

This species is a widely used medicinal plant species worldwide, such as in Iran, Saudi Arabia, and China [1,2,3]. In Iran, *J. sabina* is used as a folk medicine for abortion [1]. In China, various parts of *J. sabina*, including its leaves, berries, and aerial components, have been traditionally utilized to treat a range of ailments such as colds, dysentery, fever, rheumatoid arthritis (RA), tuberculosis, urinary infections, and urticaria. These traditional applications are supported by its diverse pharmacological properties, including antitumor [1], antidiabetic [4], antioxidant [5], hepatoprotective, and nephroprotective effects [3]. Furthermore, phytochemical analyses have identified terpenoids, lignans, flavonoids, coumarins, and other compounds as the key bioactive constituents in *J. sabina*.

This paper compiles research literature on *J. sabina* L. from 1953 to 2024, offering a systematic summary of its botanical characteristics, traditional uses, phytochemistry, pharmacological activities, and toxicity. Moreover, this work seeks to bridge the existing gaps in current research by providing a thorough and unbiased analysis of *J. sabina*. The goal is to establish a comprehensive reference that will not only serve as a valuable resource for future studies but also guide its informed and effective use across diverse applications.

## 2. Botanical Characterization

*J. sabina* typically grows in forests or thickets on rocky mountain slopes and sand dunes. It is widely found in regions such as Iran [1], Mongolia [5], Kazakhstan [6], Russia [7], Europe [8], and China [6]. Today, it is also commonly cultivated as an ornamental plant throughout China [9].

**Figure 1:**
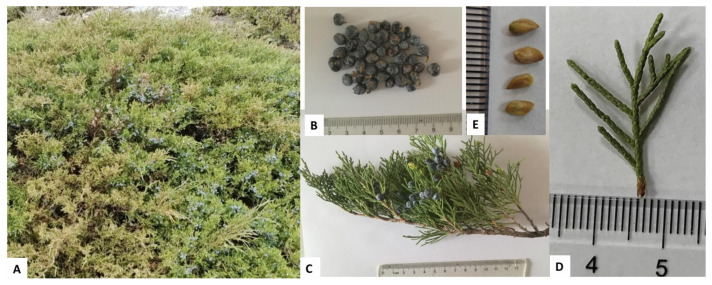
Whole plant (**A**), berries (**B**), female branches (**C**), leaves (**D**) and seeds (**E**) of *J. sabina*.

*J. sabina* (Figure 1) is a small, evergreen, creeping shrub, occasionally growing as a small tree less than 1 m in height, with grayish-brown bark. The branchlets are densely arranged, ascending, and slender, measuring about 0.8~1 mm in diameter. The leaves vary in form, with some being needle-shaped and others scale-like. Needle-like leaves are generally found on young plants and rarely on mature specimens. The scale-like leaves are decussate opposite, rhombic or rhombic-ovate in shape, ranging from 1~2.5 mm in length. These leaves feature a prominent, elliptic glandular body located in the abaxial center.

This species is primarily dioecious, though occasionally monoecious. The pollen cones are ellipsoid or oblong, measuring 3~4 mm in diameter. The seed cones are usually irregularly spherical, measuring 5~8 mm by 5~9 mm, and they transition from light brownish-green to brown, purplish-blue, or black when ripe, often coated with a white powdery substance. Each cone typically contains one or two seeds, which are ovoid, slightly flattened, 4~5 mm in diameter, and have a blunt or slightly pointed tip [2] (pp. 359).

## 3. Traditional Uses

*J. sabina* has long been utilized as a traditional ethnomedicine in various countries to treat a range of ailments, including fever, inflammation, dysentery, eczema, cancer, diabetes, skin infections, and even to induce abortion [1,6,10]. In Saudi Arabia, the aerial parts of *J. sabina* is served as folk medicine in abortion, diuresis, emetic, hypnosis, and stimulation [3]. In Iran, *J. sabina* is commonly employed in folk medicine for its abortifacient, hypnotic, diuretic, anti-ulcer, anti-rheumatic, anticonvulsant, and anti-diarrheal properties [4]. In Turkey, it is traditionally used as an antihelmintic, diuretic, and digestive remedy [11]. In the realm of traditional Chinese medicine, the fruits of *J. sabina* are valued for their abilities to dispel cold, promote meridians, warm the middle and disperse qi, facilitate diuresis, aid in purulent discharge, and promote muscle growth. These properties make the fruit effective in treating rheumatic arthritis, poor urination, windward tearing, and blurred vision [12] (p. 204). Additionally, the branches and leaves of *J. sabina* are used in folk medicine for the treatment of RA [13].

In China, the fruits of *J. sabina*, known locally as Xinjiangyuanbaishi, have a long history of use in Uyghur medicine. Their medicinal properties were first documented in the ancient text Zhu Yi Dian, published during the Song Dynasty in the early 11th century, which discusses their origin, medicinal qualities, and functional applications [14]. Another classic text, Yao Wu Zi Yuan, dating back to 1904 during the Qing Dynasty, provides detailed descriptions of the fruits’ name, morphology, origin, harvesting, processing, properties, therapeutic indications, and uses [14] (p. 377).

At present, all fruits of *J. sabina* for clinical usage come from the wild. Despite *J. sabina*’s well established clinical applications, the market contains various sources of these medicinal fruits, including *J. pseudosabina* Fisch. & C. A. Mey., *Platycladus orientalis* (L.) Franco, and *J. sibirica* Burgsd [15]. This diversity of sources underscores the importance of careful sourcing to ensure the efficacy and authenticity of the medicinal preparations.

## 4. Phytochemistry

**Figure 2:**
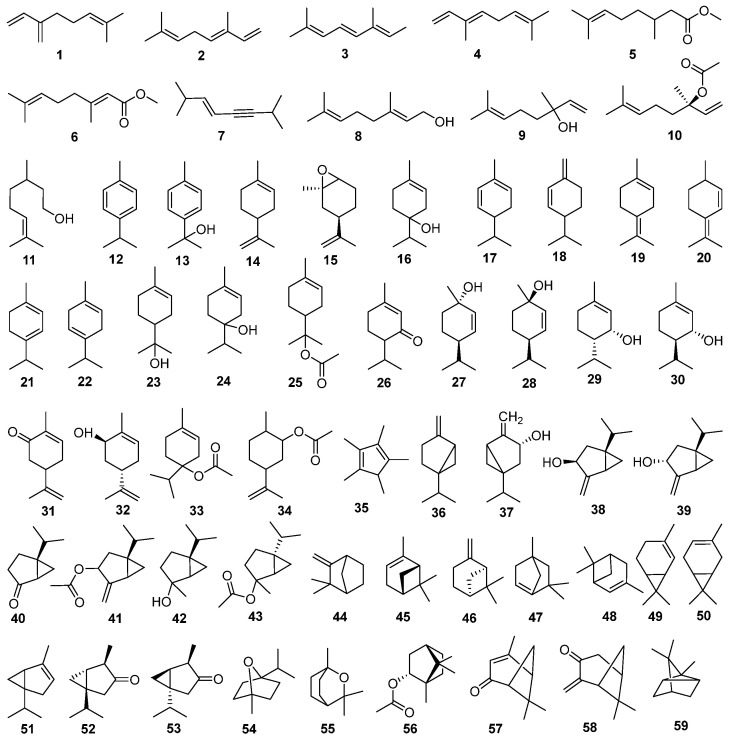
Structures of monoterpenes from *J. sabina*.

**Figure 3:**
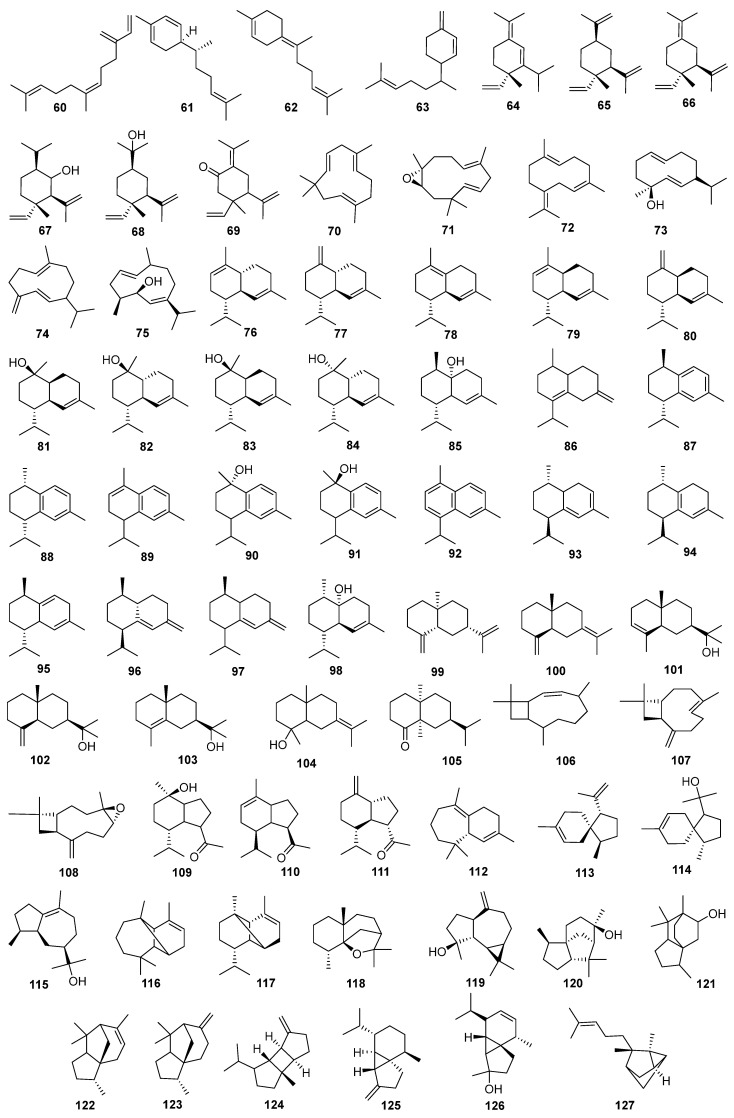
Structures of sesquiterpenes from *J. sabina*.

**Figure 4:**
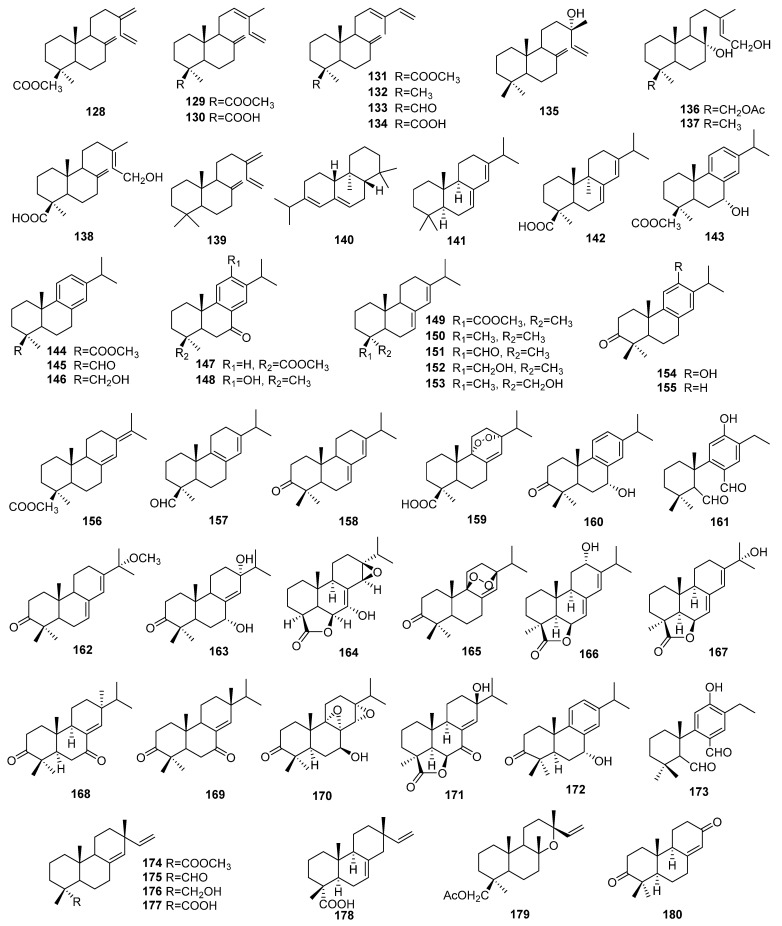
Structures of sesquiterpenes from *J. sabina*.

**Figure 5:**
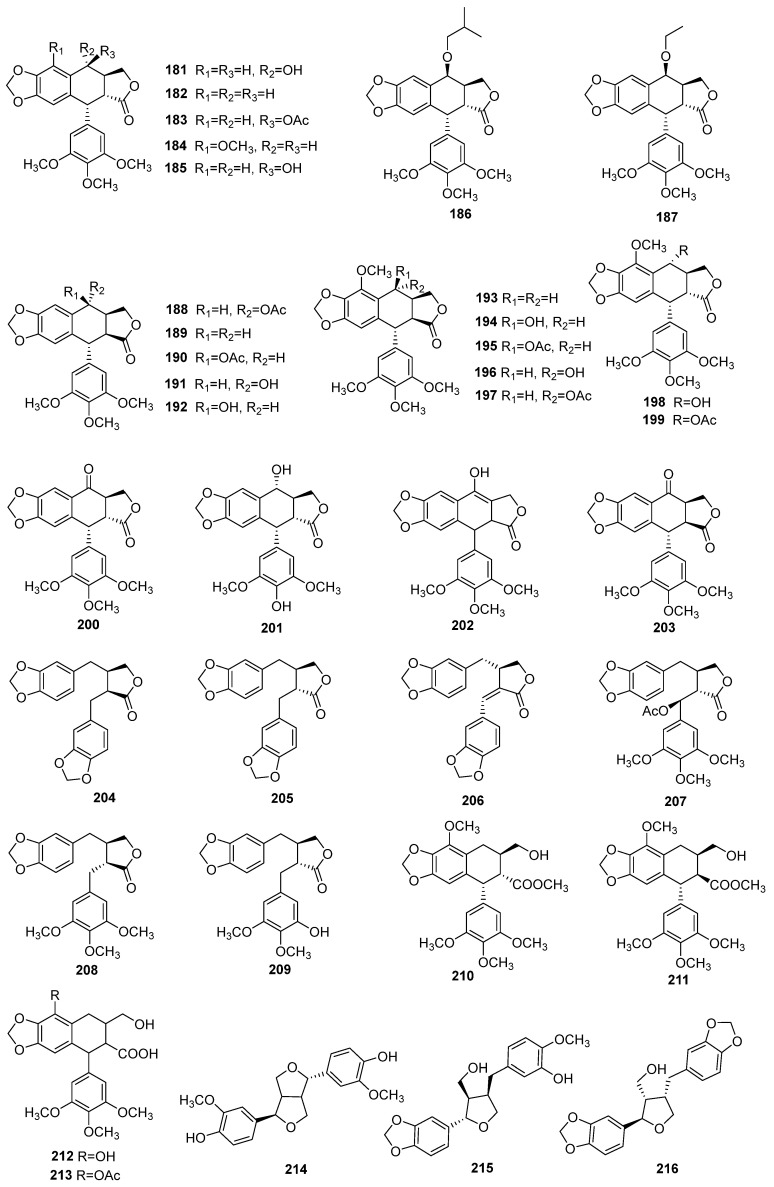
Structures of lignans from *J. sabina*.

**Figure 6:**
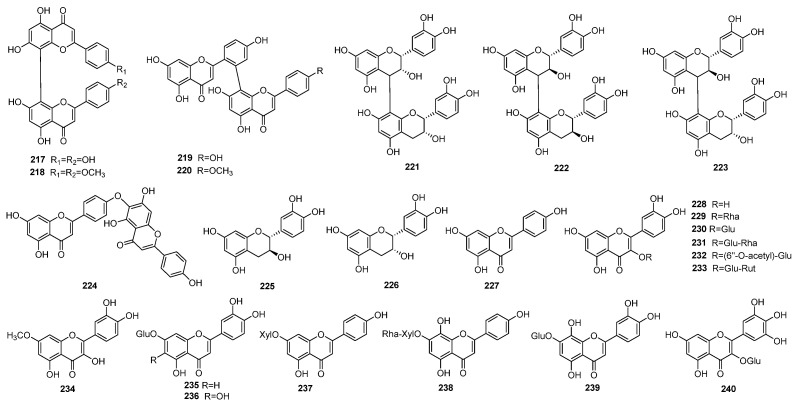
Structures of flavonoids from *J. sabina*.

**Figure 7:**
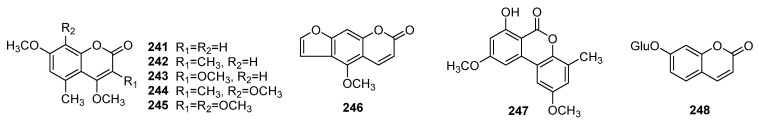
Structures of coumarins from *J. sabina*.

**Figure 8:**
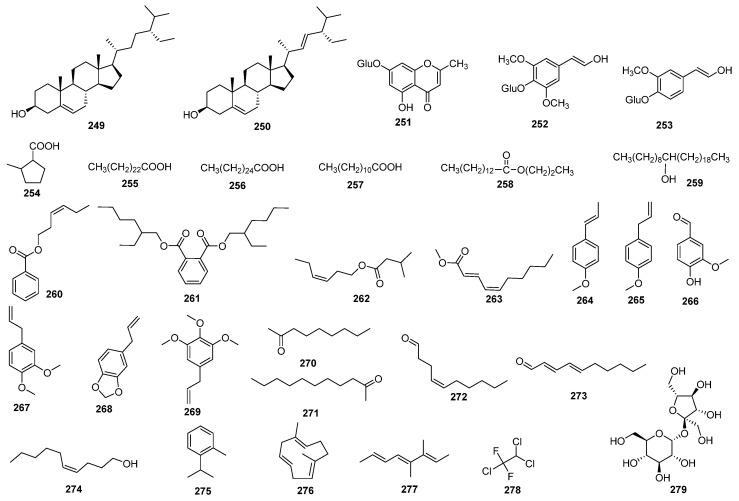
Structures of other compounds from *J. sabina*.

The exploration of the chemical composition of *J. sabina* began in 1953 [16]. Since then, 279 compounds have been identified from this plant, including 180 terpenoids, 28 lignans, 24 flavonoids, 8 coumarins, and various other compounds. Among these, terpenoids, lignans, and flavonoids are recognized as the principal bioactive components. Detailed listings of these chemical constituents can be found in Table 1, Table 2, Table 3, Table 4, Table 5, Table 6 and Table 7, with their respective structures illustrated in Figure 2, Figure 3, Figure 4, Figure 5, Figure 6, Figure 7 and Figure 8.

### 4.1. Terpenoids

Terpenoids, characterized by their core isoprene units, are widely distributed in plants and represent the most abundant class of compounds in *J. sabina*. These terpenoids can be further categorized into monoterpenes, sesquiterpenes, and diterpenes based on their structural differences.

#### 4.1.1. Monoterpenes

Monoterpenes are synthesized via the mevalonic acid pathway through the condensation of two isoprene units (C_5_H_8_). To date, 59 monoterpenes have been isolated from *J. sabina*. Based on their carbon skeleton, these monoterpenes are classified into 3 groups: 11 open-chain monoterpenes (compounds **1**–**11**), 24 monocyclic monoterpenes (compounds **12**–**35**), and 23 bicyclic monoterpenes (compounds **36**–**59**). These compounds are detailed in Table 1, and their molecular structures are presented in Figure 2. Notably, among these monoterpenes, sabinene (compound **36**) is identified as the most prevalent. And had the highest content, reaching up to 80%, significantly higher than *J. thurifera* L., *J. pygmaea* C. Koch., *J. communis* L., *J. sibirica* Burgsd., *J. excelsa* M. Bieb., and *J. oxycedrus* L. [8,17]. In addition, the content of sabinene in the fruit is higher than that in aerial parts [7].

**Table 1:** Monoterpenes isolated from *J. sabina*.

NO.	Name	Molecular Formula	Part of Plant	Reference
**1**	myrcene	C_10_H_16_	berries, leaves, stems and branches, aerial parts	[7,17,18,19]
**2**	*cis*-cimene	C_10_H_16_	berries, leaves, aerial parts	[7,18]
**3**	1,3,6-octatriene,3,7-dimethyl (alloocimene)	C_10_H_16_	leaves	[20]
**4**	alloocimene-2	C_10_H_16_	leaves	[21]
**5**	methyl citronellate	C_11_H_20_O_2_	berries, leaves, aerial parts	[5,7,18,19]
**6**	methyl geranate	C_11_H_18_O_2_	leaves, stems and branches	[20,22]
**7**	3-octen-5-yne,2,7-dimethyl-,(*E*)-	C_10_H_16_	leaves	[20]
**8**	geraniol	C_10_H_18_O	leaves, aerial parts	[19,23]
**9**	linalool	C_10_H_18_O	berries, leaves, aerial parts	[7,17,18,23]
**10**	linalyl acetate	C_12_H_20_O_2_	berries, leaves, aerial parts, stems and branches	[5,7,18,22,23]
**11**	citronellol	C_10_H_20_O	berries, leaves, aerial parts	[7,18,19,23]
**12**	*p*-cymene	C_10_H_14_	berries, leaves, aerial parts, stems and branches	[5,7,17,18,22]
**13**	*p*-cymen-8-ol	C_10_H_14_O	berries	[17]
**14**	limonene	C_10_H_16_	berries, leaves, stems and branches, aerial parts	[7,17,18,19,22]
**15**	limomene oxide	C_10_H_16_O	aerial parts	[23]
**16**	terpinen-4-ol	C_10_H_18_O	berries, aerial parts	[7,17]
**17**	*α*-phellandrene	C_10_H_16_	leaves, stems and branches	[20,22]
**18**	*β*-phellandrene	C_10_H_16_	leaves	[24]
**19**	terpinolene	C_10_H_16_	berries, leaves	[5,17,18]
**20**	isoterpinolene	C_10_H_16_	berries, leaves	[4]
**21**	*α*-terpinene	C_10_H_16_	berries, leaves, aerial parts, stems and branches	[17,18,19,22]
**22**	*γ*-terpinene	C_10_H_16_	berries, leaves, stems and branches, aerial parts	[5,17,18,22]
**23**	*α*-terpineol	C_10_H_18_O	berries, aerial parts	[17,23]
**24**	4-terpineol	C_10_H_18_O	berries, leaves	[5,18,19]
**25**	terpineol acetate	C_12_H_20_O_2_	stems and branches, aerial parts, leaves	[22,23,25]
**26**	piperitone	C_10_H_16_O	leaves	[24]
**27**	*cis*-*p*-menth-2-en-1-ol	C_10_H_18_O	leaves	[24,25]
**28**	*trans*-*p*-menth-2-en-1-ol	C_10_H_18_O	leaves	[25]
**29**	*cis*-piperitol	C_10_H_18_O	leaves	[24]
**30**	*trans*-piperitol	C_10_H_18_O	leaves	[24]
**31**	carvone	C_10_H_14_O	aerial parts	[23]
**32**	*trans*-carveol	C_10_H_16_O	aerial parts	[23]
**33**	3-cyclohexen-1-ol,4-methyl-1-(1-methylethyl)-	C_12_H_20_O_2_	leaves	[21]
**34**	dihydrocarvyl acetate	C_12_H_20_O_2_	stems and branches	[22]
**35**	1,3-cyclopentadiene,1,2,3,4,5-pentamethyl-	C_10_H_16_	leaves	[20]
**36**	rpinolene	C_10_H_16_	berries, leaves, aerial parts, stems and branches	[7,17,18,19,22,26]
**37**	sabinal	C_10_H_16_O	twigs and leaves	[22]
**38**	sabinol	C_10_H_16_O	berries, leaves, stems and branches	[17,19,22]
**39**	*trans*-sabinol	C_10_H_16_O	aerial parts	[23]
**40**	sabina ketone	C_9_H_14_O	leaves	[25]
**41**	sabinyl acetate	C_12_H_18_O_2_	berries, leaves	[5,17,19,20]
**42**	*cis*-sabinene hydrate	C_10_H_18_O	berries, leaves, aerial parts	[5,7,18,23]
**43**	*trans*-sabinene hydrate acetate	C_12_H_20_O_2_	aerial parts	[23,24]
**44**	camphene	C_10_H_16_	berries, leaves	[17,19]
**45**	*α*-pinene	C_10_H_16_	berries, leaves, stems and branches, aerial parts	[5,7,18,19,22]
**46**	*β*-pinene	C_10_H_16_	berries, leaves, aerial parts	[5,7,18,20]
**47**	*α*-fenchene	C_10_H_16_	leaves	[24]
**48**	orthodene	C_10_H_16_	leaves	[20]
**49**	*δ*-2-carene	C_10_H_16_	berries, leaves, aerial parts	[5,7,18]
**50**	*δ*-3-carene	C_10_H_16_	berries, leaves	[5,18,20]
**51**	*α*-thujene	C_10_H_16_	leaves, berries, aerial parts	[5,7,18,19,23]
**52**	*α*-thujone	C_10_H_16_O	berries, leaves, aerial parts, stems and branches	[5,7,18,19,22]
**53**	*β*-thujone	C_10_H_16_O	leaves	[19,20]
**54**	1,4-cineole	C_10_H_18_O	berries, leaves	[5,18]
**55**	1,8-cineole	C_10_H_18_O	berries, leaves	[17,19]
**56**	bornyl acetate	C_12_H_20_O_2_	berries, leaves	[27,28]
**57**	verbenone	C_10_H_14_O	aerial parts	[23]
**58**	pinocarvone	C_10_H_14_O	aerial parts	[23]
**59**	tricyclene	C_10_H_16_	leaves	[25]

#### 4.1.2. Sesquiterpenes

To date, 69 sesquiterpenes (compounds **60**–**127**) have been identified in *J. sabina*, with their details shown in Table 2 and structures depicted in Figure 3. These sesquiterpenes can be further subdivided into various categories: open-chain sesquiterpenes (compound **60**), monocyclic sesquiterpenes (compounds **61**–**75**), bicyclic sesquiterpenes (compounds **76**–**115**), and tricyclic sesquiterpenes (compounds **116**–**127**). Among them, several subtypes have been identified, including three sweet myrrhanes (compounds **61**–**63**), six elemanes (compounds **64**–**69**), two humulenes (compounds **70**–**71**), and four germacranes (compounds **72**–**75**). Additionally, the plant contains twenty-three cadinenes (compounds **76**–**98**), seven eudesmanes (compounds **99**–**105**), three caryophyllanes (compounds **106**–**108**), three oplopananes (compounds **109**–**111**), one himachalene (compound **112**), two acoranes (compounds **113**–**114**), and one guaiane sesquiterpene (compound **115**). A study has shown that the content of sesquiterpenes in the fruit is lower than that in the aerial parts [7].

**Table 2:** Sesquiterpenes isolated from *J. sabina*.

NO.	Name	Molecular Formula	Part of Plant	Reference
**60**	(*Z*)-*β*-farnesene	C_15_H_24_	leaves	[20]
**61**	zingiberene	C_15_H_24_	leaves	[20]
**62**	*β*-bisabolene	C_15_H_24_	leaves	[20]
**63**	*β*-sesquiphellandrene	C_15_H_24_	leaves	[20]
**64**	*α*-elemene	C_15_H_24_	aerial parts	[23]
**65**	*β*-elemene	C_15_H_24_	berries, leaves, aerial parts	[7,18]
**66**	*γ*-elemene	C_15_H_24_	aerial parts	[7]
**67**	shyobunol	C_15_H_26_O	aerial parts, leaves, berries	[7,24]
**68**	elemol	C_15_H_26_O	leaves, aerial parts, berries	[7,19]
**69**	*cis*-*β*-elemenone	C_15_H_22_O	berries, leaves	[18]
**70**	*α*-humulene	C_15_H_24_	berries, leaves, aerial parts	[5,7,18,23]
**71**	humulene oxide	C_15_H_24_O	aerial parts	[23]
**72**	germacrene B	C_15_H_24_	berries, leaves	[17,24]
**73**	(–)-germacra-1(10),5(*E*)-dien-4*β*-ol	C_14_H_24_O	leaves	[29]
**74**	germacrene D	C_15_H_24_	berries, leaves, aerial parts	[5,7,18]
**75**	germacrene-D-4-ol	C_15_H_26_O	berries, leaves, aerial parts	[5,7,18]
**76**	*α*-cadinene	C_15_H_24_	berries, aerial parts, leaves	[7,17,19,23]
**77**	*γ*-cadinene	C_15_H_24_	berries, leaves, aerial parts	[5,7,17,18]
**78**	*δ*-cadinene	C_15_H_24_	berries, leaves, aerial parts	[5,7,18,20]
**79**	*α*-muurolene	C_15_H_24_	berries, leaves, aerial parts	[5,7,18,23]
**80**	*γ*-muurolene	C_15_H_24_	berries, aerial parts	[7,17]
**81**	*τ*-muurolol	C_15_H_26_O	aerial parts, leaves	[7,25]
**82**	*α*-cadinol	C_15_H_26_O	berries, aerial parts, leaves	[7,17,24]
**83**	*δ*-cadinol	C_15_H_26_O	berries, leaves	[5,18,19]
**84**	*τ*-cadinol	C_15_H_26_O	berries, leaves	[5,18,25]
**85**	*epi*-cubenol	C_15_H_26_O	aerial parts	[30]
**86**	bicyclo[4.4.0]dec-1-ene, 2-isopropyl-5-methyl-9-methylene	C_15_H_24_	leaves	[20]
**87**	*trans*-calamenene	C_15_H_22_	aerial parts	[30]
**88**	*cis*-calamenene	C_15_H_22_	aerial parts	[23]
**89**	*α*-calacorene	C_15_H_20_	aerial parts	[23]
**90**	calamenene-10*β*-ol	C_15_H_22_O	aerial parts	[23,30]
**91**	calamenene-10*α*-ol	C_15_H_22_O	aerial parts	[23,30]
**92**	cadalene	C_15_H_18_	berries, aerial parts	[23,30]
**93**	*trans*-muurola-3,5-diene	C_15_H_24_	leaves	[24]
**94**	*trans*-cadina-1(6),4-diene	C_15_H_24_	leaves	[24]
**95**	*trans*-cadina-1,4-diene	C_15_H_24_	leaves	[24]
**96**	*cis*-muurola-4(14),5-diene	C_15_H_24_	leaves	[24]
**97**	*trans*-murrola-4(14),5-diene	C_15_H_24_	leaves	[24]
**98**	1,10-di-epi-cubenol	C_15_H_26_O	leaves	[24]
**99**	*β*-selinene	C_15_H_24_	berries	[17]
**100**	*γ*-selinene	C_15_H_24_	berries	[17]
**101**	*α*-eudesmol	C_15_H_26_O	aerial parts	[7]
**102**	*β*-eudesmol	C_15_H_26_O	leaves, aerial parts	[7,19]
**103**	*γ*-eudesmol	C_15_H_26_O	leaves, aerial parts	[7,19]
**104**	juniper camphor	C_15_H_26_O	leaves	[4]
**105**	valeranone	C_15_H_26_O	leaves	[4]
**106**	*cis*-caryophyllene	C_15_H_26_	berries	[17]
**107**	*β*-caryophyllene	C_15_H_24_	berries, leaves, stems and branches	[7,18,22,23]
**108**	caryophyllene oxide	C_15_H_24_O	aerial parts	[23]
**109**	oplopanone	C_15_H_26_O_2_	berries, leaves	[29,31]
**110**	*α*-oplopenone	C_15_H_24_O	leaves	[24]
**111**	*β*-oplopenone	C_15_H_24_O	berries, leaves, aerial parts	[7,18]
**112**	*β*-himachalene	C_15_H_24_	leaves	[20]
**113**	(–)-*β*-acoradiene	C_15_H_24_	leaves	[20]
**114**	*α*-acorenol	C_15_H_26_O	leaves	[25]
**115**	bulnesol	C_15_H_26_O	leaves	[24]
**116**	*α*-longipinene	C_15_H_24_	leaves	[20]
**117**	*α*-copaene	C_15_H_24_	leaves	[20]
**118**	*cis*-dihydroagarofuran	C_15_H_26_O	aerial parts	[7]
**119**	spathulenol	C_15_H_24_O	aerial parts	[7]
**120**	cedrol	C_15_H_26_O	berries, leaves, stems and branches	[5,18,20,22]
**121**	allo-cedrol	C_15_H_26_O	leaves	[24]
**122**	*α*-cedrene	C_15_H_24_	leaves	[25]
**123**	*β*-cedrene	C_15_H_24_	leaves	[25]
**124**	*β*-bourbonene	C_15_H_24_	leaves	[24]
**125**	*β*-cubebene	C_15_H_24_	leaves	[24]
**126**	*epi*-cubebol	C_15_H_24_O	leaves	[24]
**127**	santalene	C_15_H_24_	leaves	[20]

#### 4.1.3. Diterpenes

A total of 53 diterpenes (compounds **128**–**180**) were identified in the berries, leaves, and aerial parts of *J. sabina* (as shown in Table 3 and Figure 4). These diterpenes are categorized into 4 main groups: 12 labdane diterpenes (compounds **128**–**139**), 34 abietane diterpenes (compounds **140**–**173**), 6 pimarane diterpenes (compounds **174**–**179**), and 1 podocarpane diterpene (compound **180**). Among these, the abietane diterpenes are the most prominent constituents.

**Table 3:** Diterpenes isolated from *J. sabina*.

NO.	Name	Molecular Formula	Part of Plant	Reference
**128**	methyl myrcecommunate	C_21_H_32_O_2_	berries	[31]
**129**	methyl *cis*-communate	C_21_H_32_O_2_	berries	[31]
**130**	*cis*-communic acid	C_20_H_30_O_2_	bark	[32]
**131**	methyl *trans*-communate	C_21_H_32_O_2_	berries	[31]
**132**	*trans*-biformene	C_20_H_32_	berries	[31]
**133**	*trans*-communal	C_20_H_30_O	berries	[31]
**134**	*trans*-communic acid	C_20_H_30_O_2_	twigs and leaves, berries, bark	[32,33,34]
**135**	manool	C_20_H_34_O	aerial parts	[30]
**136**	19-acetoxy-labd-13(*E*)-en-8,15-diol	C_22_H_38_O_4_	leaves	[29]
**137**	labd-*E*-13-ene-8,15-diol	C_20_H_36_O_2_	leaves, berries	[6,31]
**138**	isocupressic acid	C_20_H_32_O_3_	leaves, bark	[32,35]
**139**	sclarene	C_20_H_32_	aerial parts	[7]
**140**	abietatriene	C_20_H_30_	leaves	[24]
**141**	abietadiene	C_20_H_32_	aerial parts, leaves, berries	[7,24]
**142**	4-*epi*-abietic acid	C_20_H_30_O_2_	leaves, aerial parts, twigs and leaves	[29,30,36]
**143**	methyl 7*α*-hydroxycallitrisate	C_21_H_30_O_3_	berries	[31]
**144**	methyl callitrisate	C_21_H_30_O_2_	berries	[31]
**145**	4-*epi*-dehydroabietal	C_20_H_28_O	berries	[31]
**146**	4-*epi*-dehydroabietol	C_20_H_30_O	berries	[31,34]
**147**	methyl 7-oxo-callitrisate	C_21_H_28_O_3_	berries	[31]
**148**	sugiol	C_20_H_28_O_2_	seeds, bark	[32]
**149**	4-*epi*-abietate	C_31_H_32_O_2_	berries	[31]
**150**	abieta-7,13-diene	C_20_H_32_	berries	[31]
**151**	4-*epi*-abietal	C_20_H_30_O	berries, leaves, aerial parts	[7,25,29,31]
**152**	4-*epi*-abietol	C_20_H_32_O	berries	[31]
**153**	abietol	C_20_H_32_O	berries	[31]
**154**	hinokione	C_20_H_28_O_2_	berries	[37]
**155**	abieta-7,11,13-trien-3-one	C_20_H_28_O	berries	[31]
**156**	4-*epi*-neoabietate	C_21_H_32_O_2_	berries	[31]
**157**	4-*epi*-palustral	C_20_H_30_O	berries	[31]
**158**	abieta-7,13-dien-3-one	C_20_H_30_O	berries, aerial parts, leaves	[7,29,31]
**159**	4-epi-palustric acid 9a,13α-endoperoxide	C_20_H_30_O_4_	leaves	[29]
**160**	7*α*-hydroabieta-8,11,13-triene-3-one	C_20_H_28_O_2_	berries	[34]
**161**	12-hydroxy-6,7-secoagieta-8,11,13-triene-6,7-dial	C_19_H_26_O_3_	berries	[34]
**162**	15-methoxy-abieta-7,13-dien-3-one	C_21_H_32_O_2_	berries	[34]
**163**	7,13-dihydroxy-8(14)-abieten-3-one	C_20_H_32_O_3_	berries	[34]
**164**	13*β*,14*β*-epoxya12-hydroxy biet-7-en-19,6*β*-olide	C_19_H_26_O_4_	berries	[34]
**165**	9*β*,13*β*-endoperoxide-8(14)-abieten-3-one	C_20_H_30_O_3_	berries	[34]
**166**	sabiperone A	C_20_H_28_O_3_	aerial parts	[6]
**167**	sabiperone B	C_20_H_28_O_3_	aerial parts	[6]
**168**	sabiperone D	C_21_H_32_O_2_	aerial parts	[6]
**169**	sabiperone E	C_21_H_32_O_2_	aerial parts	[6]
**170**	sabiperone F	C_20_H_30_O_4_	aerial parts	[6]
**171**	juniperolide	C_20_H_28_O_4_	aerial parts	[6]
**172**	3*β*,7*α*-dihydroxy-abieta-8,11,13-triene	C_20_H_28_O_2_	aerial parts	[6]
**173**	12-hydroxy-6,7-secoabieta-8,11,13-triene-6,7-dial	C_19_H_26_O_3_	bark	[28]
**174**	methyl sandaracopimarate	C_21_H_32_O_2_	berries	[31]
**175**	sandaracopimaral	C_20_H_30_O	berries	[31]
**176**	sandaracopimarol	C_20_H_32_O	berries	[31]
**177**	sandaracopimaric acid	C_20_H_30_O_2_	aerial parts, bark	[30,32]
**178**	isopimaric acid	C_20_H_30_O_2_	aerial parts	[30]
**179**	19-acetoxy-13-epimanoyl oxide	C_22_H_36_O_3_	berries	[34]
**180**	sabiperone C	C_17_H_24_O_2_	aerial parts	[6]

### 4.2. Lignans

Lignans are among the representative bioactive components of *J. sabina*, with podophyllotoxin analogs being the primary structural types. To date, 28 lignans have been reported from this species (Table 4 and Figure 5). The first discovery of podophyllotoxin (compound **181**) in *J. sabina* dates back to 1953, along with savinin (compound **206**), which was also extracted from the dried needles of the plant [16]. Podophyllotoxin is known for its diverse biological activities, including anticancer [16], antiviral [38], and insecticidal properties [39]. Following this discovery, numerous podophyllotoxin analogs have been identified in *J. sabina*. Additionally, both bicyclic lignans and monocyclic lignans have been observed in the plant’s phytochemical profile. Another study explored the accumulation and formation mechanisms of podophyllotoxins in-depth [40]. The leaves have a higher content than other parts, and the content of the lignans in different parts shows inconsistent trends with increasing age [41].

**Table 4:** Lignans isolated from *J. sabina*.

NO.	Name	Molecular Formula	Part of Plant	Reference
**181**	podophyllotoxin	C_22_H_22_O_8_	fresh leaves, twigs and leaves, berries	[33,37,42,43]
**182**	deoxypodophyllotoxin	C_22_H_22_O_7_	leaves and stems, berries, leaves, seeds	[27,37,43,44,45]
**183**	acetyl epipodophyllotoxin	C_24_H_24_O_9_	leaves, berries	[37,44]
**184**	*β*-peltatin A methyl ether	C_23_H_24_O_8_	aerial parts, leaves, bark, berries	[32,37,46,47]
**185**	epipodophyllotoxin	C_22_H_22_O_8_	aerial parts	[47]
**186**	epipodophyllotoxin isobutyl ether	C_26_H_30_O_8_	berries	[37]
**187**	epipodophyllotoxin ethyl ether	C_24_H_26_O_8_	berries, aerial parts	[37,47]
**188**	acetyl-picropodophyllotoxin	C_24_H_24_O_9_	berries	[48]
**189**	deoxypicropodophyllotoxin	C_22_H_22_O_7_	leaves, berries	[37,44,48]
**190**	acetyl epipicropodophyllotoxin	C_24_H_24_O_9_	leaves	[44]
**191**	picropodophyllotoxin	C_22_H_22_O_8_	berries, leaves, leaves and stems	[37,45,46,48]
**192**	epipicropodophyllotoxin	C_22_H_22_O_8_	leaves	[44]
**193**	*β*-peltatin B methyl ether	C_23_H_24_O_8_	aerial parts, leaves	[46,47]
**194**	2′-methoxy epipicropodophyllotoxin	C_23_H_24_O_9_	leaves	[46]
**195**	2′-methoxyepipicropodophyllotoxin acetate	C_25_H_26_O_10_	leaves	[46]
**196**	2′-methoxypicropodophyllotoxin	C_23_H_24_O_9_	leaves	[46]
**197**	2′-methoxypicropodophyllotoxin acetate	C_25_H_26_O_10_	leaves	[46]
**198**	2′-methoxypodophyllotoxin	C_23_H_24_O_9_	leaves	[46]
**199**	2′-methoxypodophyllotoxin acetate	C_25_H_26_O_10_	leaves	[46]
**200**	podophyllotoxone	C_22_H_20_O_8_	leaves	[48]
**201**	4′-demethylpodophyllotoxin	C_21_H_20_O_8_	berries	[37]
**202**	dehydropodophyllotoxin	C_22_H_20_O_8_	leaves, berries	[37,49]
**203**	picropodophyllotoxone	C_22_H_20_O_8_	leaves	[49]
**204**	savinin	C_20_H_18_O_6_	needles	[16]
**205**	(–)-deoxypodorhizon	C_20_H_18_O_6_	leaves	[44]
**206**	(–)-hibalactone	C_20_H_16_O_6_	berries	[37]
**207**	podorhizol acetatlie	C_24_H_26_O_9_	leaves	[46]
**208**	yatein	C_22_H_24_O_7_	berries	[37]
**209**	(–)-3-O-demethylyatein	C_21_H_22_O_7_	leaves, aerial parts, berries	[37,47,49]
**210**	sabinaperin A	C_24_H_28_O_9_	aerial parts	[47]
**211**	sabinaperin B	C_24_H_28_O_9_	aerial parts	[47]
**212**	junaphtoic acid	C_22_H_24_O_9_	leaves	[49]
**213**	junaphtoic acid acetate	C_24_H_26_O_10_	leaves	[49]
**214**	(+)-epipinoresinol	C_20_H_22_O_6_	leaves	[49]
**215**	epoxylignans	C_20_H_22_O_6_	leaves	[48]
**216**	(–)-dihydrosesamin	C_20_H_20_O_6_	aerial parts	[47]

### 4.3. Flavonoids

Flavonoids are another significant group of natural compounds found in *J. sabina*. Research has shown that the leaves of *J. sabina* have the highest total flavonoid content, measured at 31.53 ± 1.29 mg·g^−1^ of extracts, expressed as quercetin equivalent [10]. In total, 24 flavonoid compounds have been identified in the seeds, stems, leaves, and twigs of this species (listed in Table 5 and illustrated in Figure 6). These compounds include six flavones, eight flavonols, two flavanes, and eight biflavones. The flavones and flavonols are typically present in glycoside forms. Notably, in flavones, the glycosides are linked at the C-7 position, whereas in flavonols, they are connected at the C-3 position.

Biflavones are the most abundant flavonoids in *J. sabina* and are widely distributed within the Cupressaceae family. Based on their linkage patterns, biflavones are categorized into two types: C-C-linked and C-O-C-linked biflavones. All biflavonones in *J. sabina*, except for hinokiflavone (compound **224**), are formed by the linkage of two flavonoid moieties through a carbon–carbon bond.

Tian et al. (2006) have further investigated the flavonoid content in different parts of *J. sabina*, revealing that the branches contain the lowest flavonoid levels, while the fruits have the highest concentration. Their study also suggests that the peak flavonoid content in the fruit occurs at the end of October, whereas the highest concentration in the leaves is observed in April [50].

Chen (2017) surveyed the content of amentoflavone (**219**) in *J. sabina* leaves from different areas and times. The highest content of amentoflavone (**219**) appears in July, and the content of amentoflavone (**219**) in *J. sabina* leaves from different regions also varies [13]. This indicated that the content of components depends on the phenological phases of plant development and the environment.

**Table 5:** Flavonoids isolated from *J. sabina*.

NO.	Name	Molecular Formula	Part of Plant	Reference
**217**	cupressuflavone	C_30_H_18_O_10_	seeds, leaves	[27,51]
**218**	cupressuflavone 4′,4′′′-di-methylether	C_32_H_22_O_10_	leaves, berries, seeds	[27,37,51,52]
**219**	amentoflavone	C_30_H_18_O_10_	twigs and leaves, leaves and berries, berries	[33,37,51,52]
**220**	podocarpusflavone A	C_31_H_20_O_10_	twigs and leaves, berries	[37,53]
**221**	procyanidin B2	C_30_H_26_O_12_	stems, bark	[32,54]
**222**	procyanidin B3	C_30_H_26_O_125_	stems, bark	[32,54]
**223**	procyanidin B4	C_30_H_26_O_12_	stems, fresh leaves	[42,54]
**224**	hinokiflavone	C_30_H_18_O_10_	twigs and leaves, berries	[34,54]
**225**	(+)-catechin	C_15_H_14_O_6_	leaves, stems, berries	[37,38,54]
**226**	(–)-epicatechin	C_15_H_14_O_6_	stems	[54]
**227**	apigenin	C_15_H_10_O_5_	leaves, twigs and leaves, needles	[42,53,55]
**228**	quercetin	C_15_H_10_O_7_	leaves, twigs and leaves	[38,51,53]
**229**	quercetin-3-O-*α*-L-rhamnofuranoside	C_21_H_20_O_11_	fresh leaves, needles	[51,55]
**230**	isoquercetin	C_21_H_20_O_12_	twigs and leaves, needles	[38,55]
**231**	quercetin 3-O-(6-O-*β*-rhamosyl-*β*-D-glucopyranoside)	C_26_H_18_O_15_	needles	[55]
**232**	quercetin-3-O-(6″-O-acetyl)-*β*-D-glucopyranoside	C_23_H_22_O_13_	fresh leaves	[51]
**233**	rutin	C_27_H_30_O_16_	fresh leaves	[38]
**234**	rhamnetin	C_16_H_12_O_7_	leaves	[50]
**235**	luteolin 7-O-*β*-D-glucoside	C_21_H_20_O_11_	twigs and leaves	[53]
**236**	6-hydroxy-luteolin-7-O-*β*-D-glucoside	C_21_H_20_O_12_	seeds	[27]
**237**	isoscutellarein 7-O-*β*-D-xylopyranoside	C_20_H_18_O_9_	fresh leaves	[38]
**238**	isoscutellarein 7-O-*β*-D-xylopyranose-(1→3)-*α*-L-rhamnoside	C_26_H_28_O_14_	twigs and leaves	[38]
**239**	hypolaetin 7-O-*β*-xylopyranoside	C_21_H_20_O_12_	fresh leaves, twigs and leaves	[36,56]
**240**	myricetin 3-O-*β*-D-glucoside	C_21_H_20_O_13_	fresh leaves	[51]

### 4.4. Coumarins

Coumarins are notable constituents of *J. sabina*, characterized by their core benzopyrone structure. Eight coumarin compounds (compounds **241**–**248**) have been isolated from various parts of *J. sabina*, including its berries, leaves, seeds, and bark. Among these, only one coumarin compound, skimmianine (compound **248**), is identified as a glucoside, which is extracted from the fresh leaves of the plant. The details of these coumarin components are presented in Table 6, with their chemical structures illustrated in Figure 7.

**Table 6:** Coumarins isolated from *J. sabina*.

NO.	Name	Molecular Formula	Part of Plant	Reference
**241**	siderin	C_12_H_12_O_4_	leaves, seeds	[27,52]
**242**	coumarsabin	C_13_H_14_O_4_	leaves, berries, seeds	[27,34,35,52]
**243**	3-methoxy coumarsabin	C_14_H_16_O_5_	berries	[34]
**244**	8-methoxy coumarsabin	C_14_H_16_O_5_	leaves, berries	[34,52]
**245**	3,8-dimethoxy coumarsabin	C_14_H_16_O_6_	berries	[37]
**246**	bergaptin	C_12_H_8_O_4_	bark	[32]
**247**	sabilactone	C_16_H_14_O_5_	bark	[32]
**248**	skimmin	C_15_H_16_O_8_	fresh leaves	[35]

### 4.5. Other Compounds

Beyond the aforementioned groups of compounds, *J. sabina* contains a variety of other chemical constituents, including steroids, chromones, phenylethanol glycosides, fatty acids and esters, small molecular weight alcohols, and other minor components. These compounds are displayed in Table 7, with their corresponding structures depicted in Figure 8.

**Table 7:** Other compounds isolated from *J. sabina*.

NO.	Name	Molecular Formula	Part of Plant	Reference
**249**	*β*-sitosterol	C_29_H_50_O	twigs and leaves, seeds, bark, berries	[27,31,32,33,36]
**250**	stigmasterol	C_29_H_48_O	berries	[37]
**251**	undulatoside A	C_16_H_18_O_9_	fresh leaves	[51]
**252**	syringin	C_17_H_24_O_9_	fresh leaves	[51]
**253**	coniferin	C_16_H_22_O_8_	twigs and leaves	[53]
**254**	2-methyl-3-cyclopentanecarboxylic acid	C_7_H_12_O_2_	leaves	[20]
**255**	lignoceric acid	C_24_H_48_O_2_	bark	[32]
**256**	cerotic acid	C_26_H_52_O_2_	bark	[32]
**257**	dodecanoic acid	C_12_H_24_O_2_	aerial parts	[23]
**258**	propionic acetate myristic acid	C_17_H_34_O_2_	twigs and leaves, seeds	[27,53]
**259**	10-nonacosanol	C_29_H_60_O	berries	[31]
**260**	(*Z*)-3-hexenyl benzoate	C_13_H_16_O_2_	leaves	[24]
**261**	1,2-benzenedicarboxylic acid, bis (2-ethylhexyl) ester	C_24_H_38_O_4_	leaves	[21]
**262**	(*Z*)-3-Hexenyl 3-methylbutyrate	C_11_H_18_O_2_	leaves	[20]
**263**	methyl *trans*-2-*cis*-4-decadienoate	C_11_H_20_O_2_	leaves	[24]
**264**	anethole	C_10_H_12_O	leaves	[19]
**265**	estragole	C_10_H_12_O	leaves	[19]
**266**	vanillin	C_8_H_8_O_3_	stems and branches	[22]
**267**	methyl eugenol	C_11_H_14_O_2_	berries, leaves	[18]
**268**	safrole	C_10_H_10_O_2_	leaves	[24]
**269**	elemicine	C_12_H_16_O_3_	leaves	[24]
**270**	2-nonanone	C_9_H_18_O	leaves	[25]
**271**	2-undecanone	C_11_H_22_O	aerial parts	[7]
**272**	(*Z*)-4-decenal	C_10_H_18_O	leaves	[24]
**273**	(*E*, *E*)-2,4-decadienal	C_10_H_16_O	leaves	[24]
**274**	*Z*-4-decen-1-ol	C_10_H_20_O	leaves	[24]
**275**	benzene,1-methyl-2-(1-methylethyl) –	C_10_H_14_	leaves	[20]
**276**	pregeijerene B	C_12_H_18_	leaves	[24]
**277**	2,4,6-octatriene,3,4-dimethyl	C_10_H_16_	leaves	[20]
**278**	ethane,1,2,2-trichloro-1,1-difluoro	C_2_HCl_3_F_2_	leaves	[21]
**279**	sucrose	C_12_H_22_O_11_	twigs and leaves, seeds	[27,53]

## 5. Pharmacology

The essential oils, extracts, and isolated compounds from *J. sabina* exhibit a wide range of biological activities, including insecticidal, antimicrobial, antioxidant, anti-inflammatory, antitumor, hepatoprotective, and nephroprotective effects.

### 5.1. Insecticidal Effect

Research conducted by Li (2006) has demonstrated that the essential oils and pure compounds derived from *J. sabina* possess significant insecticidal properties [37]. The essential oils are found to be effective against adults of *Musca domestica* Vicial and *Sitophilus zeamais* Molschulsky, as well as against third-instar larvae of armyworms. Additionally, lignans and coumarins extracted from the plant show antifeedant activity against armyworm larvae and toxic effects against *Plutella xylostella* L. Li’s study has further revealed that specific structural features, such as different substituent groups at the 4*β* position and the degree of unsaturation in the C-ring of podophyllotoxin derivatives, can significantly impact their insecticidal potency.

Moreover, acetone extracts from the fruits of *J. sabina* have demonstrated significant antifeedant, stomach toxicity, and contact toxicity effects against *Pieris rapae* L. These extracts also show strong antifeedant and stomach toxic activities against *Mythimna separata* Walker, although the effects are weaker against *Plutella xylostella* L. Additionally, the acetone extracts are found to inhibit the population growth of *Sitophilus zeamais* Motschulsky and *Tribolium castaneum* Herbst, as well as disrupt the development of *Helicoverpa armigera* Hubner. The essential oils derived from *J. sabina* fruits exhibit a potent fumigant effect on *P. xylostella* L., *S. zeamais* Motschulsky, and *T. castaneum* Herbst [57].

Subsequent studies have shown that essential oils from *J. sabina* possess contact-killing, fumigation, avoidance, and sublethal effects against *Paratrioza sinica* [58,59]. It is suggested that the insecticidal activity may be associated with the inhibition of Na-K-ATPase activity [59]. Further research highlights the insecticidal efficacy of terpinen-4-ol (compound **16**), which demonstrates activity against various pests, including *P. xylostella* L., *M. separata* Walker, *Heliothis armigera* Hubner, *Musca domestica* L., *S. zeamais*, *T. castaneum*, *Trialeurodes vaporariorum*, and *Tetranychus urticae* Koch [60].

**Figure 9:**
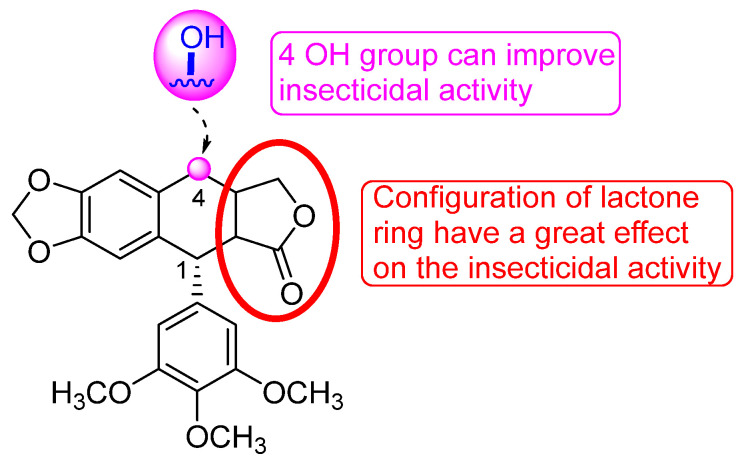
Studies of lignans with insecticidal activity.

The petroleum ether extract of *J. sabina* has also demonstrated insecticidal activity against the fifth-instar larvae of *Pieris rapae* L. Additionally, the lignans deoxypodophyllotoxin (compound **182**), deoxypicropodophyllotoxin (compound **189**), and podophyllotoxin (compound **181**) are investigated for their insecticidal activities and structure–activity relationships (Figure 9). All these lignans show significant insecticidal effects, with the trans-lactone ring being identified as a crucial functional group contributing to their bioactivity [39]. In a subsequent study, podophyllotoxin (compound **181**) exhibits antifeedant and toxic activities, as well as a marked growth inhibitory effect on *Plutella xylostella* [61]. Further research has found that the insecticidal activity of deoxypodophyllotoxin (compound **182**) is due to its ability to inhibit the activity of metabolic enzymes in *Mythimna separata* Walker [62].

In summary, the essential oils, extracts, and several pure compounds derived from *J. sabina* exhibit substantial insecticidal activities. Among these, podophylloids are considered the primary insecticidal active components, while terpenes are recognized as the major insecticidal constituents in essential oils.

### 5.2. Antimicrobial Activity

Lignans extracted from the leaves of *J. sabina* have been evaluated for their antiviral properties. Their activities are tested against Herpes simplex virus type 1 (HSV-1) infecting monkey kidney fibroblasts (CV-1), and vesicular stomatitis virus (VSV) infecting hamster kidney fibroblasts (BHK). The study has found that lignans containing a trans-tetralinelactone unit exhibit more potent antiviral effects [63].

Several studies have also indicated that the volatile oils extracted from different parts of *J. sabina* possess significant antimicrobial properties. These volatile oils have been shown to effectively inhibit a variety of microorganisms, including *Escherichia coli*, *Bacillus cereus*, *Staphylococcus aureus*, *Pseudomonas aeruginosa*, *Candida albicans*, *Clostridium perfringens*, *Fusarium verticillioides*, and *F. graminearum* [8,18,64]. Furthermore, research has demonstrated that the volatile oils derived from the branches and leaves of *J. sabina* show inhibitory effects on *Aspergillus niger* and various yeast strains. It is also observed that the volatile oils from *J. sabina* harvested between August and September exhibit the most robust antibacterial activity [65].

Extracts from the leaves of *J. sabina* have demonstrated the ability to inhibit the growth of various microorganisms, including *Bacillus subtilis*, *Escherichia coli*, *Staphylococcus aureus*, *Bacillus thuringiensis*, *Aerogenic intestinal* stem, *Proteus vulgaris*, yeast, *Aspergillus niger*, *Penicillium*, and *Rhizopus* [66]. Additionally, Song et al. have reported that the leaves of *J. sabina* can inhibit *Mycobacterium tuberculosis*, a finding that is further verified in clinical settings [67]. Another study has confirmed that methanolic and ethanolic extracts of *J. sabina* effectively suppress the reproduction of bacterial strains such as *E. coli* O157, *Salmonella typhimurium*, *Bacillus cereus* (ATCC 7064), and *Staphylococcus aureus* [68].

A study demonstrated that 15-year-old *J. sabina* roots exhibited the best characteristics of inhibiting the reproduction of Methicillin-resistant *Staphylococcus aureus* [41].

In summary, the volatile oils and extracts derived from *J. sabina* have demonstrated potent antibacterial, antifungal, and antiviral activities. Future research should focus on screening other compounds for antimicrobial activity and elucidating the underlying mechanisms of these effects.

### 5.3. Antioxidant Effect

To assess the antioxidant effects of *J. sabina*, various assays have been utilized, including DPPH radical scavenging, ferrous ion-chelating, superoxide anion radical scavenging, and ferric-reducing antioxidant power (FRAP) assays. The extracts, essential oils, polyphenols, total flavonoids, and pure compounds derived from *J. sabina* have all been shown to exhibit significant antioxidant activities. Given its potential as an oxidative inhibitor, *J. sabina* holds promising prospects for future development.

The ethyl acetate and butanol fractions of *J. sabina* exhibit particularly strong antioxidative capacity, which is positively correlated with their flavonoid content. Specific flavonoids such as catechin (compound **225**), cupressuflavone (compound **217**), cupressuflavone 4′,4‴-dimethylether (compound **218**), amentoflavone (compound **219**), podocarpusflavone A (compound **220**), quercetin (compound **228**), rutin (compound **233**), isoscutellarein 7-O-β-D-xylopyranoside (compound **237**), and myricetin 3-O-β-D-glucoside (compound **240**) are demonstrated to possess DPPH free radical scavenging abilities. Notably, catechin (compound **225**) and quercetin (compound **228**) show more potent antioxidant effects compared to vitamin C, indicating their potent antioxidative potential [50]. The aforementioned outcome suggested that the relationship between the structure of flavonoids and antioxidant activity indicates the following: (a) the antioxidant activity of flavonoids is superior to that of biflavonoids; (b) the number of hydroxyl groups have a significant impact on antioxidant activity; and (c) the hydroxy group at position C-3 has a great effect on anti-inflammatory activity (compound **228** vs. **233**).

However, another study has indicated that the essential oils extracted from the male and female leaves, as well as the fruits of *J. sabina*, exhibit varying degrees of antioxidant activity, as determined by the DPPH assay, deoxyribose degradation test, and a modified deoxyribose assay [4].

Both aqueous and ethanol extracts from the leaves and fruits of *J. sabina* are evaluated for their antioxidant activity. All extracts demonstrate the ability to scavenge DPPH radicals and reduce ferric ions. The aqueous extract of the fruits, in particular, displays ferrous ion-chelating capacity. Furthermore, all extracts, except for the ethanol extract of the fruits, show the ability to scavenge superoxide anion radicals [10]. Li et al. have further explored the antioxidant properties of polyphenols extracted from *J. sabina* using the aforementioned four methods, confirming that these polyphenols possess vigorous antioxidant activities [69]. Additionally, both methanolic and ethanolic extracts of *J. sabina* are found to effectively reduce DPPH radicals, with IC_50_ values of 19.70 and 0.33 μg·mL^−1^, respectively [68].

Moreover, several studies have shown that the total flavonoids from *J. sabina* exhibit significant antioxidant effects [56,70].

### 5.4. Antitumor Effect

Extensive research has demonstrated that *J. sabina* exhibits significant antitumor properties. San et al. have evaluated the antitumor effects of 19 cyclolignans isolated from the leaves of *J. sabina* against several cancer cell lines, including P-388 murine leukemia, A-549 human lung carcinoma, and HT-29 colon carcinoma. Among these, deoxypodophyllotoxin and β-peltatin A methyl ether show the most potent activities, with IC_50_ values ranging from 2.5 to 4 ng·mL^−1^ [63].

In vitro experiments have further demonstrated that 80% ethanol extracts from the fruits and branchlets of both male and female *J. sabina* can inhibit the proliferation of Hela, MDA-MB-468, and KB cells [1,71]. Another study has revealed that the ethanol extract of *J. sabina* fruits exhibits lower IC_50_ values against CHO (29.2 μg·mL^−1^), HepG2 (1.2 μg·mL^−1^), and SKOV3 (0.8 μg·mL^−1^) cell lines compared to normal rat fibroblasts (27.3 μg·mL^−1^) [72].

Huyan et al. have found that aqueous extracts of *J. sabina* can inhibit the growth of HepG2 and K562 cells by blocking the G0/G1 phase of the tumor cell cycle, interfering with cell adhesion, and increasing the levels of FasL, caspase 3, and caspase 9 [73]. Additionally, Kavaz et al. have reported that methanolic and ethanolic extracts of *J. sabina* are effective in suppressing the proliferation of MCF-7 and MDA-MB-231 cell lines [68].

### 5.5. Anti-Inflammatory Effect

Zhao et al. have discovered that the total flavonoids extracted from *J. sabina* possess therapeutic effects against RA by inhibiting the production of tumor necrosis factor-alpha (TNF-α) and interleukin 1 beta (IL-1β) cytokines in a rat model induced by complete Freund’s adjuvant. Furthermore, these flavonoids significantly reduce arthritis scores, synovial hyperplasia, and inflammatory cell infiltration [38].

In addition, the total flavonoids from *J. sabina* are found to effectively suppress auricle swelling in mice, reduce peritoneal capillary permeability response in mice, and decrease cotton ball-induced granuloma formation in rats. They also reduce egg white and carrageenan-induced toe swelling in rats [74]. Zhu et al. have reported that the total flavonoids, along with quercetin and isoquercetin isolated from *J. sabina*, exhibit varying degrees of inhibitory effects on COX-1/2, 5-LO, and TNF-α in RAW264.7 cells stimulated by lipopolysaccharide (LPS) [75].

In summary, total flavonoids, quercetin, and isoquercetin derived from *J. sabina* show significant potential as anti-inflammatory agents.

### 5.6. Abortion Effect

*J. sabina* has traditionally been used as an abortifacient. Pages et al. have reported that the volatile oil of *J. sabina* induces miscarriage, primarily due to the presence of sabinyl acetate (compound **171**) [28]. However, the specific mechanisms and additional substances involved in this effect require further investigation.

### 5.7. Analgesic Effect

Maitituersun has studied the analgesic effects of the total flavonoids from *J. sabina*. The findings reveal that these flavonoids can significantly reduce the number of writhing reactions induced by acetic acid in mice and increase the pain threshold on a hot plate test [74].

### 5.8. Immunoregulatory Effect

The total flavonoids of *J. sabina* and the pure compound cedrol (compound **120**) have demonstrated potent immunoregulatory effects. Cedrol, in particular, has been identified as a novel neutrophil agonist [74,76]. Nonetheless, the detailed pharmacological mechanisms underlying these effects remain unknown and warrant further research.

### 5.9. Hepatoprotective and Nephroprotective Effects

Extracts from the aerial parts of *J. sabina* have been demonstrated to protect against carbon tetrachloride (CCl_4_)-induced liver toxicity [5]. In a subsequent study, the petroleum ether fraction of this plant is found to significantly reduce the levels of aspartate aminotransferase (AST), bilirubin, and alanine aminotransferase (ALT), indicating its hepatoprotective properties. Additionally, pure compounds such as epi-cubenol (compound **85**), manool (compound **135**), and 4-epi-abietic acid (compound **142**) have been reported to have potent hepatoprotective effects [30].

### 5.10. Lipid-Lowering Effect

An in vivo study has demonstrated that polyphenols derived from *J. sabina* can reduce body weight in hyperlipidemic rats, lower triglycerides, total cholesterol, and low-density lipoprotein cholesterol levels, while increasing high-density lipoprotein cholesterol levels after administration [76]. These findings indicate that polyphenols from *J. sabina* have the potential to lower blood lipid levels.

### 5.11. Cholinesterase Inhibitory Effect

Cholinesterases, including acetylcholinesterase (AChE) and butyrylcholinesterase (BChE), are evaluated for inhibition by various extracts of *J. sabina*. The aqueous extract of ripe fruits and leaves shows no inhibitory effect on AChE. Meanwhile, ethanol extracts from unripe fruits, ripe fruits, and leaves exhibit only weak inhibitory effects on AChE. Regarding BChE, all extracts demonstrate weak inhibition [11]. Further investigation indicates that podophyllotoxin plays a significant role in BChE inhibition, while AChE inhibition shows a strong correlation with deoxypodophyllotoxin [77].

The effects of cholinesterase inhibition of different ages and tissues of *J. sabina* were further evaluated. Fifteen-year-old *J. sabina* leaves exhibited the best characteristics of AChE. And 7-year-old *J. sabina* exhibited the best characteristics of BChE [41].

### 5.12. Antidiabetic Effect

Low-density lipoprotein is highly susceptible to oxidation in diabetic patients, and glycated hemoglobin, along with insulin, serves as a marker for blood glucose control. Asgary et al. [4] have demonstrated that the volatile oils from *J. sabina* fruits, as well as from the male and female branches and leaves, can inhibit hemoglobin glycosylation, insulin glycosylation, and oxidative stress. These findings suggest that these volatile oils have potential applications in developing hypoglycemic drugs [3]. Further studies show that with the increase in *J. sabina*, the antidiabetic effect gradually decreases [41].

### 5.13. Antifertility Effect

Li et al. have investigated the antifertility effects of *J. sabina* fruits on male rats and found that the fruits significantly reduce male fertility. The study suggests that this effect may be due to damage to the epididymis function without altering androgen levels [78]. Further studies have indicated that podophyllum compounds are associated with antifertility effects, with the pure compound podophyllotoxin (compound **181**) likely contributing to this effect through the TNF-*α* and caspase signaling pathways [43,79].

### 5.14. Hemostasis Effect

A traditional remedy involving soaking *J. sabina* leaves with a small amount of white sugar has been used to treat epistaxis (nosebleeds). This treatment is later confirmed in clinical practice. The mechanism underlying its effectiveness in treating epistaxis may be related to the anti-inflammatory properties of flavonoids and tannins, which help relieve smooth muscle spasms, improve microcirculation, dispel pathogenic factors, and promote healing [80].

### 5.15. Other Activities

The bioflavone cupressuflavone (compound **217**), isolated from *J. sabina*, has been observed to reduce motor activity, coordination, and balance in rats, indicating potential effects on the central nervous system [81].

## 6. Toxicology

Toxicological research primarily focuses on evaluating the risks associated with substances, potential biological exposure, and human health, encompassing acute toxicity tests, chronic toxicity tests, tumor induction tests, genotoxicity tests, and ecological toxicity tests. It has been reported that pregnant mice subcutaneously injected with volatile oil from *J. sabina* leaves between the 6th and 15th days of pregnancy experience significant weight loss compared to the control group by the 19th day of pregnancy. Hepatotoxicity is observed in pregnant mice; however, no toxicity is detected in the fetuses [28].

There are few clinical research reports on the toxicity of *J. sabina*. Volatile oil has local irritants, and long-term use on the skin and mucous membranes can cause severe inflammation [13]. Therefore, it is not for oral administration. Shi et al. have assessed the acute toxicity of the volatile oil from *J. sabina* over a 14-day period. The data indicate that mice die within 12 to 24 h after gavage, with an LD_50_ of 2570 mg·kg^−1^. Additionally, the volatile oil demonstrates clear subchronic toxicity [82]. Another study has revealed that the volatile oil of *J. sabina* can induce sperm abnormalities in mice and increase the proportion of micronuclei in myelocytes [83]. Shi has further suggested that the maximum no-effect level of the volatile oil should be below 6.9 mg/kg·bw·day [83].

Other studies have shown that the LD_50_ values of the volatile oil from the aerial parts of *J. sabina* are 15 times its effective dosage, suggesting a relatively safe range for the use of the volatile oil from the aerial parts of the plant [30].

## 7. Conclusions and Perspectives

This paper provides a comprehensive summary of the studies on the botanical characteristics, traditional uses, phytochemistry, biological activities, and toxicity profile of *J. sabina*. Phytochemical investigations have identified 279 secondary metabolites isolated from *J. sabina*, predominantly terpenoids, lignans, flavonoids, coumarins, and other compounds. The studies reveal that these compounds vary in type and content across different parts of the plant. Most phytochemical research on *J. sabina* has concentrated on its essential oils, which are primarily composed of monoterpenes, sesquiterpenes, diterpenes, alkanes, small molecular organic acids, and small molecular alcohols. These are commonly analyzed using gas chromatography-mass spectrometry (GC/MS), HPLC/MS, and HPLC-DAD-ESI-TQ-MS/MS.

Phytochemical studies have also highlighted that flavonoids and lignans are the key components in *J. sabina*. Lignans, particularly podophyllotoxin, have demonstrated significant insecticidal, anticholinesterase, and antifertility activities. Biflavonoids, a crucial class of flavonoids, exhibit a range of pharmacological effects. These insights suggest that biflavonoids and podophyllum compounds can serve as quality markers (Q-markers) for *J. sabina*, guiding future research and development.

Essential oils, various extracts, and pure compounds derived from *J. sabina* have demonstrated a wide range of biological activities, including insecticidal, antimicrobial, antioxidant, anti-inflammatory, and cytotoxic effects. However, several challenges remain unaddressed. First, many pharmacological activities have been confirmed only through in vitro experiments, lacking in vivo validation. Second, the mechanisms underlying some pharmacological effects have not been thoroughly elucidated. Third, the pharmacological activities and mechanisms of action of pure compounds have not been comprehensively studied. Fourth, there has been limited modern pharmacological exploration of the traditional uses of *J. sabina*.

To address these gaps, future research should focus on investigating pharmacological activities in vivo, elucidating deep-seated mechanisms of action, identifying specific molecular targets, and isolating active pharmacological substances. Additionally, expanding the scope of *J. sabina*’s efficacy to treat more diseases using a variety of modern scientific methods is essential.

Moreover, there are limited reports regarding the clinical application of *J. sabina*. To ensure the safety and efficacy of its clinical use, well-designed clinical trials are needed to confirm its therapeutic potential and establish safe usage guidelines.

In summary, the available evidence indicates that *J. sabina* is not only a medicinal plant with a broad range of therapeutic applications but also a highly valuable insecticidal plant. As a traditional medicinal plant, *J. sabina* holds significant potential in treating various human diseases and promoting health. Although considerable progress has been made in understanding its chemical composition and pharmacological activities, the research on its mechanisms of action, specific targets, and active pharmacological substances remains insufficient. Moreover, there is limited exploration of its traditional therapeutic uses through modern pharmacological methods, particularly in understanding the relationship between traditional efficacy and active components. Additionally, there is a scarcity of studies evaluating the clinical effectiveness and safety of *J. sabina*. Therefore, it is worth performing further studies in clinical practice and establishing quality control standards.

## Data Availability

No new data were created or analyzed in this study.
